# Ocular ultrasonography of sea turtles

**DOI:** 10.1186/s13028-020-00551-1

**Published:** 2020-09-10

**Authors:** Caterina Muramoto, Vinícius Cardoso-Brito, Ana Cláudia Raposo, Thais Torres Pires, Arianne Pontes Oriá

**Affiliations:** 1 School of Veterinary Medicine and Zootechny, Federal University of Bahia, Avenida Adhemar de Barros, 500, Ondina, Salvador, BA 40170-110 Brazil; 2 Fundação Pró-Tamar, Rua Rubens Guelli 134/ 307, Itaigara, Salvador, Bahia 41815-135 Brazil

**Keywords:** *Caretta caretta*, *Chelonia mydas*, *Eretmochelys imbricate*, Eye, *Lepidochelys olivacea*, Ultrasound

## Abstract

**Background:**

Environmental changes contribute to the development of ophthalmic diseases in sea turtles, but information on their eye biometrics is scarce. The aim of this study was to describe ophthalmic ultrasonographic features of four different sea turtle species; *Caretta caretta* (Loggerhead turtle; n = 10), *Chelonia mydas* (Green turtle; n = 8), *Eretmochelys imbricata* (Hawksbill turtle; n = 8) and *Lepidochelys olivacea* (Olive ridley; n = 6) under human care. Corneal thickness, scleral ossicle width and thickness, anterior chamber depth, axial length of the lens, vitreous chamber depth and axial globe length were measured by B-mode sonography with a linear transducer. Carapace size and animal weight were recorded. A sonographic description of the eye structures was established.

**Results:**

The four species presented an ovate eyeball, a relatively thin cornea, and a small-sized lens positioned rostrally in the eye bulb, near the cornea, resulting in a shallow anterior chamber. The scleral ossicles did not prevent the evaluation of intraocular structures, even with a rotated eye or closed eyelids; image formation beyond the ossicles and measurements of all proposed structures were possible. B-mode sonography was easily performed in all animals studied. The sonographic characteristics of the eye were similar among the four species. Since there was a correlation between the size of the eye structures and the size of the individual, especially its carapace size, the differences found between *E. imbricata* and *Caretta caretta* are believed to be due to their overall difference in size.

**Conclusions:**

Sonography is a valuable tool in ophthalmic evaluation of these species. Only minor differences were found between the species in this study, reinforcing their phylogenetic proximity and their similar functions and habitats.

## Background

Sea turtles have different methods for orientation and spatial localization [1], and vision is one of the main senses involved in environmental interactions, hunting and defence against predators [2–6]. The retina of these species changes throughout their lives to adapt to different visual stimuli, resulting from the various environments with which they are in contact, both terrestrial and pelagic, during the migration process [7]. Sea turtles can differentiate colours and, like other aquatic species, they have spherical lenses which are the main means of light refraction in water [8].

The sea turtle's eye is proportionally small relative to its body size in comparison to other vertebrates, and the pupil and lens are small relative to the eye [4]. Other characteristics of these animals' ocular structures are a high sensitivity to corneal touch, strong eyelids, the presence of a well-developed nictitating membrane, scleral ossicles, eyelid scales, retractor bulbi and pyramidalis muscles [4, 9–11], which have a protective function. However, ophthalmic diseases such as corneal ulcers, keratitis and fibropapillomas are not uncommon in these animals [10, 12–15] and can cause loss of visual function, thereby restricting the possibility of their reintroduction to, or survival in their natural habitat [16].

Anthropic action and environmental changes contribute to the development of ophthalmic diseases in these animals [13] and are the main causes for their conservation classification status as vulnerable and critically endangered species [17–20]. Among the seven known sea turtle species, five are found along the Brazilian coast: loggerhead turtle (*Caretta caretta*), hawksbill turtle (*Eretmochelys imbricata*), olive ridley turtle (*Lepidochelys olivacea*), green turtle (*Chelonia mydas*) and leatherback turtle (*Dermochelys coriacea*) [12, 17–21].

Imaging techniques, such as ultrasound, can contribute to morphological and structural assessments of the eye. Echobiometric eye evaluation has already been employed in chelonians [22, 23], but there are no such studies for sea turtle eyes. Herein we describe sonographic features of the eyes of four sea turtle species, compare the findings among them, and provide anatomical details that could be useful for personnel involved in sea turtle conservation.

## Methods

### Sea turtles

Eyes (*n* = 64) from 32 juvenile to adult sea turtles (10 *Caretta caretta*, 8 *Chelonia mydas*, 8 *Eretmochelys imbricata* and 6 *Lepidochelys olivacea*) kept at the TAMAR Project (Brazil) visitors centre (VC) were used in the study. The VC tanks fulfil the Standard Permit Conditions for Care and Maintenance of Captive Sea Turtles [24]. Physical and ophthalmic examinations were performed by the technical staff (TAMAR Project) and a veterinary ophthalmologist (UFBA). All animals were subjected to clinical evaluation, and inspection of the eye and periocular region in normal light for gross abnormalities with a 3X binocular magnifying loupe and trans illuminator. Animals that presented any clinical signs of systemic disease or gross eye or periocular abnormalities were excluded from the study.

All animals were manually restrained and body weight (BW), curved carapace length (CCL) and curved carapace width (CCW) were measured prior to ultrasonographic evaluation. Environmental temperature and humidity ranged from 23.7 to 28.9 °C and from 57 to 85%, respectively.

### Ultrasonographic evaluation

**Fig. 1:**
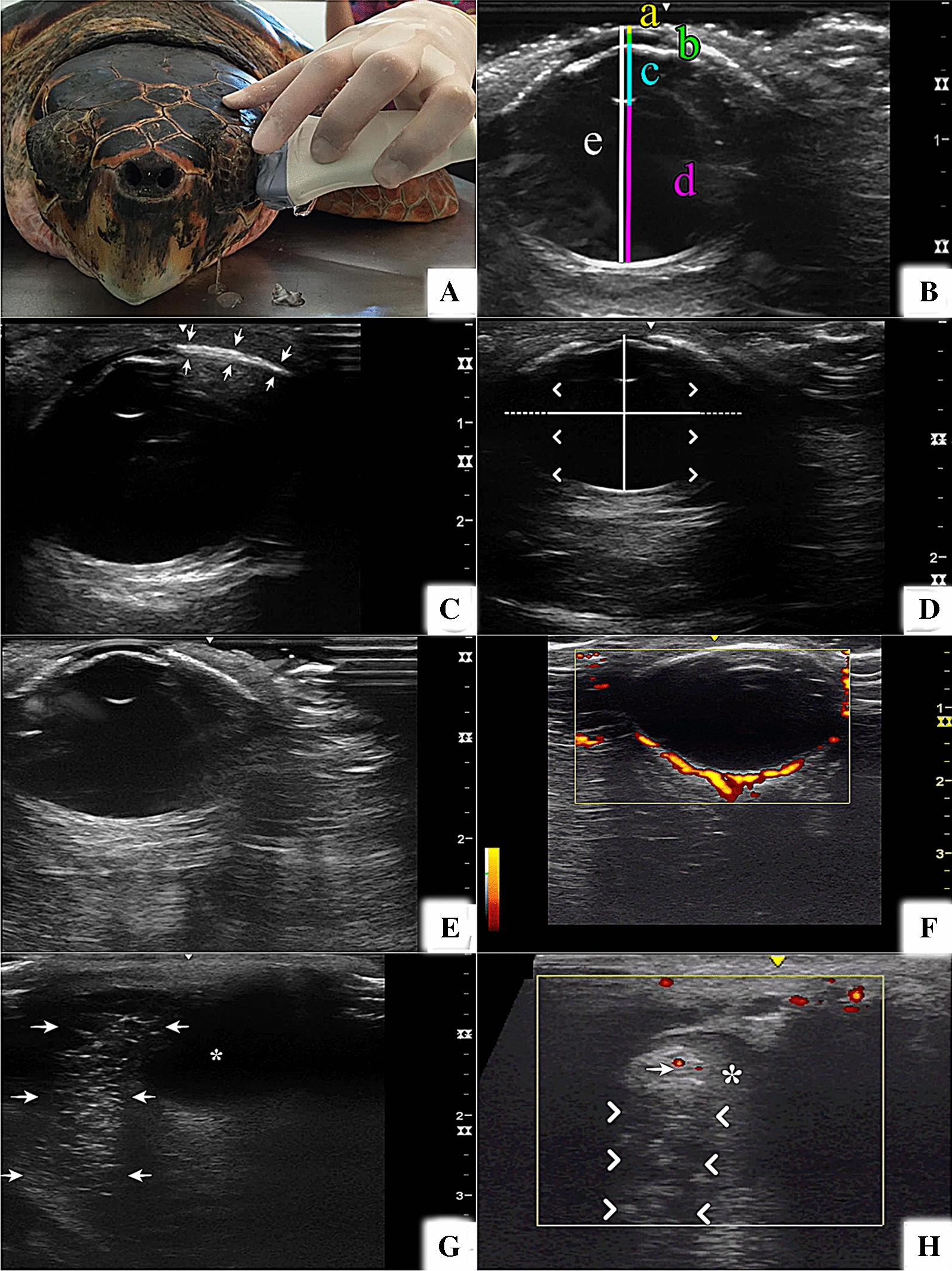
Ultrasound images of sea turtle eyes. **A** Examination of a *Caretta caretta* eye. **B** *Chelonia mydas* eye—coloured lines show the axial globe length (a), corneal thickness (b), anterior chamber depth (c), lens axial length (d), vitreous chamber depth (e). **C** *Caretta caretta* eye—slightly oblique image from which scleral ossicle width and thickness were measured (between arrows). **D** *Eretmochelys imbricata* eye showing oval shape with central optic axis (line) smaller than the equatorial diameter (dashed line), indicating the posterior shading of the orbit bone (asterisk). **E** *Lepidochelys olivacea* eye—note that despite the posterior ossicle artefacts, visualization of the posterior portions is not impeded. **F** *Caretta caretta* eye in power Doppler mode showing segments of blood vessels (in yellow–orange) distributed in the scleral cartilage. **G** Part of the salt gland (between arrows) adjacent to the bulb (*) of *Lepidochelys olivacea*. **H** *Caretta caretta* eye in power Doppler mode showing a large blood vessel (*) adjacent to the salt gland (between arrows), where few and small vessels were identified (arrow head)

Prior to ultrasonographic evaluation, one drop of topical anaesthetic (1% tetracaine hydrochloride with 0.1% phenylephrine hydrochloride, Anestesico®, Allergan, São Paulo, Brazil) was administered to each eye; this dosage was sufficient to perform the ultrasound evaluation for up to 20 min. A portable ultrasound system, Logiq-e® (GE Medical Systems, Wuxi, China), with a 7–12 MHz linear transducer was used. Among the system pre-sets, the “small/superficial parts” were chosen. The ultrasound acoustic gel (Carbogel ULT®, São Paulo, Brazil) was placed on the probe surfaces and the transducer was gently propped on either the eyelid or directly on the corneal surface (Fig. 1A). B-mode scanning of the eyeball was performed in sagittal, dorsal and oblique planes by the same investigator (CM) to minimize inter examiner measurement errors. Doppler mode scanning was used to verify the presence of vascularization in some structures, such as the retina, choroid and scleral cartilage.

At the level of the central optic axis of the eyeball, at its maximal anteroposterior axis, corneal thickness (CT), anterior chamber depth (ACD), axial length of the lens (ALL), vitreous chamber depth (VCD) and axial globe length (AGL) were measured (Fig. 1B), and by slightly shifting the transducer laterally or medially, an image of the full scleral ossicle was obtained, and scleral ossicle width and thickness (SOT) were measured (Fig. 1C). The AGL was measured from the corneal surface to the fundus, not including the thick part of the scleral cartilage that surrounds the posterior half of the bulb.

### Statistical analyses

Shapiro–Wilk normality test was used for the physical body measurements and sonographic ocular measurements. SPSS version 22.0 was used for the analysis, and the level of significance was set to 5%. Comparison of the variables between right and left eyes was performed by Wilcoxon test. Kruskal–Wallis test was used for comparison of the same variable between species. Spearman test was used for correlations among BW, CCL, AGL and ocular ultrasound variables.

## Results

The ocular ultrasound images were obtained trans-palpebrally and trans-corneally, with no difference in the image quality. It was possible to identify the bulb's oval shape, and maximum circumference was along the plane perpendicular to the central optic axis (Fig. 1D). It was possible to see almost the entire length of the bulb in all four species, but in some eyes, the extremities of the larger circumference (equatorial diameter) were covered by the posterior acoustic shadow produced by the orbital bones (Fig. 1D), preventing their accurate measurements. Spontaneous rotation of the eye bulb occurred relatively frequently and the examiner had to wait for spontaneous repositioning to obtain the image on the central optic axis.

The cornea was visualized as a convex, smooth and thin hyperechogenic double line with a hypo to anechoic area between the lines. The anterior and posterior segments were filled with homogeneous anechoic fluid. The lens had an almond-like biconvex shape, with smooth, slender, hyperechogenic anterior and posterior surfaces, and a homogeneous anechoic central portion. The ciliary body and iris were viewed together as a circular echogenic band positioned anterior to the lens equator.

The scleral ossicle was easily identified as a thin, slightly convex hyperechogenic structure that surrounds the bulb as a strip, from the corneal border (limbus) to the anterior sclera, and it could be measured (anteroposterior measurement and thickness). The scleral ossicle had a slightly coarse echotexture and, depending on the angle of insonation, it produced a discrete acoustic shadow and/or posterior reverberation. However, in both cases, the artefacts were discrete and did not prevent imaging beyond the scleral ossicles (Fig. 1E).

The scleral cartilage is a tissue present in the sclera of sea turtles, which surrounds the back of the bulb, posteriorly to the retina and choroid. It was identified as a thick and echogenic tissue with interspersed hyperechogenic foci, which gave the tissue a coarse echotexture; the scleral cartilage had an irregular posterior surface and poorly defined limits towards adjacent retrobulbar tissues and choroid. Several segments of blood vessels were identified interspersed with the scleral cartilage in some individuals using power or colour Doppler mode (Fig. 1F).

The optic nerve was observed as a homogeneous, hypoechogenic tubular structure and its insertion into the bulb could not be visualized on the image of the central optic axis. Parts of the salt gland could be evaluated and showed a rough echotexture with irregular contours and mixed echogenicity (Fig. 1G). In several individuals, a large blood vessel with slowly flowing contents adjacent to the gland, was observed. In the Doppler mode evaluation, a few small vessels were identified inside the salt gland in some individuals (Fig. 1H).

**Fig. 2:**
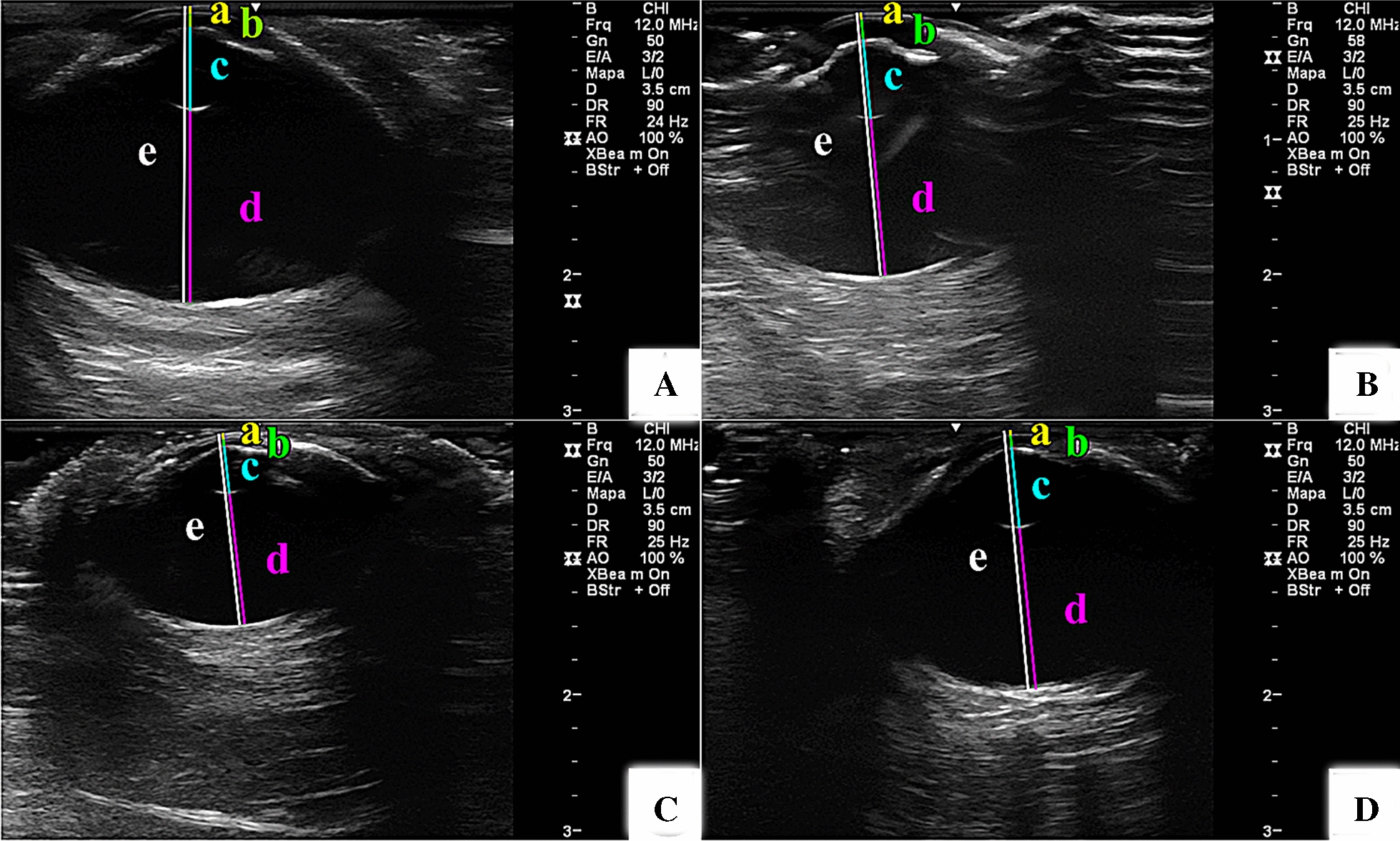
Ultrasonographic images of the eyes of four different species of sea turtle with different body weights: *Caretta caretta* (**A**), *Chelonia mydas* (**B**), *Eretmochelys imbricata* (**C**), and *Lepidochelys olivacea* (**D**), taken at the same transducer frequency and the same scale size (note the ruler to the right of each image). Similar structures at the level of the central optic axis are indicated by coloured lines as follows: corneal thickness (a), anterior segment depth (b), lens axial length (c), vitreous chamber depth (d), and axial globe length (e). Note the evident difference in size, especially the depth of the vitreous chamber and the axial length of the globe

The measured values presented a non-parametric distribution (P < 0.05). There were no significant differences between right and left eyes for any of the variables (P > 0.05). There was wide variation in the biometric data for individuals of different ages and sizes, because the inclusion method was by convenience sample (Additional file 1). There were significant differences between species when comparing the same structures (P < 0.05) (Table 1), mainly with respect to biometric measurements (Fig. 2). However, although there was a difference in the measured values of the ocular structures, the eyes did not present any significant dissimilarities. The width of the scleral ossicle was smaller for *E. imbricata* compared to the other species studied. The anterior chamber depth and ALL were significantly lower for *E. imbricata* compared to *Caretta caretta* and *L. olivacea*. The AGL was larger for *Caretta caretta* and *L. olivacea* than for the other two species. All intraocular structures were smaller in *E. imbricata* than in *Caretta caretta*. *E. imbricata* was the smallest species in terms of BW and carapace size, comprising a relatively young population.

**Table 1:** Median ± semi-interquartile interval and confidence interval of animal and ultrasound measurements in sea turtle eyes

	All animals (*n* = 32)		*Caretta caretta* (*n* = 10)		*Chelonia mydas* (*n* = 8)		*Eretmochelys imbricate* (*n* = 8)		*Lepidochelys olivacea* (*n* = 6)	
	Median ± S-IQR	CI	Median ± S-IQR	CI	Median ± S-IQR	CI	Median ± S-IQR	CI	Median ± S-IQR	CI
BW (kg)	38.6 ± 18.65	38.964–58.482	40.2 ± 41.19	12.028–123.872	31.3 ± 22.465	3.289–65.604	13.20 ± 12.8	4.633–40.1	35.0 ± 4.30	24.839–43.628
CCL (cm)	69.1 ± 12.75	64.690–73.919	73.5 ± 14.975	64.216–97.051	62.3 ± 18.63	37.183–85.384	50.5 ± 11.465	43.248–69.919	62.25 ± 5.44	56.668–68.465
CCW (cm)	63.70 ± 8.40	57.351–68.202	61.0 ± 12.66	42.528–90.722	65.1 ± 12.35	38.654–82.546	43.3 ± 10.88	28.546–69.354	61.4 ± 5.07	56.939–69.061
SOW (cm)	1.07 ± 0.085	1.057–1.117	1.04 ± 0.13^a^	0.856–1.358	0.93 ± 0.10^ab^	0.789–1.166	0.82 ± 0.09^b^	0.685–1.010	1.05 ± 0.04^a^	0.969–1.305
SOT (cm)	0.07 ± 0.01	0.068–0.078	0.08 ± 0.005^a^	0.069–0.085	0.06 ± 0.005^b^	0.056–0.074	0.07 ± 0.005^b^	0.060–0.688	0.07 ± 0.005^b^	0.0592–0.073
CT (cm)	0.04 ± 0.005	0.040–0.044	0.05 ± 0.005^a^	0.042–0.051	0.04 ± 0.005^c^	0.027–0.043	0.04 ± 0.00^bc^	0.036–0.041	0.04 ± 0.005^b^	0.041–0.051
ASD (cm)	0.1 ± 0.015	0.100–0.115	0.11 ± 0.01^ab^	0.100–0.122	0.1 ± 0.025^ab^	0.080–0.138	0.08 ± 0.005^b^	0.076–0.117	0.11 ± 0.02^a^	0.102–0.126
LAL (cm)	0.48 ± 0.07	0.449–0.495	0.44 ± 0.095^a^	0.424–0.549	0.48 ± 0.065^ab^	0.400–0.520	0.36 ± 0.045^b^	0.347–0.417	0.53 ± 0.025^a^	0.518–0.555
VCD (cm)	1.18 ± 0.125	1.118–1.218	1.25 ± 0.15^a^	1.241–1.441	1.07 ± 0.125^b^	0.898–1.140	0.97 ± 0.075^b^	0.947–1.078	1.21 ± 0.03^a^	1.195–1.248
AGL (cm)	1.79 ± 0.215	1.676–1.824	1.88 ± 0.23^a^	1.784–2.079	1.63 ± 0.18^b^	1.382–1.779	1.41 ± 0.13^b^	1.379–1.603	1.89 ± 0.045^a^	1.838–1.904

*S-IQR* semi-interquartile interval, *CI* confidence interval, *BW* body weight, *CCL* curved carapace length, *CCW* curved carapace width, *SOW* scleral ossicle width, *SOT* scleral ossicle thickness, *CT* corneal thickness, *ASD* anterior chamber depth, *LAL* lens axial length, *VCD* vitreous chamber depth, *AGL* axial globe length. Different letters in a column indicate significant difference between groups (P < 0.05)

Moderate to strong correlations (r ≥ 0.5) were found between almost all variables and BW and CCL in *Caretta caretta* and *Chelonia mydas*, except for SOT and BW in *Caretta caretta* and for SOT and CCL in both species (Additional file 2). Strong correlations (r ≥ 0.8) were seen between BW and CCL and between BW and CCW in all species, except for *L. olivacea* (r ≤ 0.3). Strong correlations (r ≥ 0.9) were present between CCW and CCL in all species. Moderate negative correlations were seen in *L. olivacea* between CCL and CT (r = −0.6; P = 0.034) and between CCL and ACD (r = −0.7; P = 0.008). Moderate positive correlations in *L. olivacea* were seen between CCL and ALL (r = 0.7; P = 0.015) and between CCL and AGL (r = 0.7; P = 0.024)*.* In general, the highest echobiometric values were found in the largest animals.

## Discussion

Several ultrasonographic studies have been performed on sea turtles to evaluate the coelomic cavity [25], but to the authors’ knowledge, there are no reports on ophthalmic echobiometry in these species. However, the increasing number of sea turtles in conservation centres [14], and considering studies on ophthalmic diseases in these species [10, 12–15], there is rising concern among institutions and staff about the conservation of vision in these animals, to promote quality of life and allow for the possibility of reintroduction into a free living environment.

Ophthalmic examination of sea turtles has been reported to be difficult to perform due to high tear film viscosity, high corneal sensitivity, strong eyelid incursion, bulbar retraction, small pupil size, and a more complicated medical pupillary dilation than in many other species, even with the use of neuromuscular blockers such as vecuronium associated with phenylephrine or atracurium associated, or not, with atropine [14]. Ultrasound is a complementary modality for the diagnosis and follow-up of ophthalmic diseases because it enables evaluating the eye structures, even in the presence of opaque media [26] such as fibropapillomatosis, keratitis, ulcerations [13] and cataracts [15].

The high-frequency (10 and 12 MHz) linear transducer allowed the evaluation of the entire sea turtle eyeball, the size of which reached about 2 cm in depth. The contact surface of the transducer was 4.3 cm, enabling a good view of the eyeball, but the orbital bone produced posterior acoustic shadowing that prevented visualization of the lateral part of the bulb and measurement of the equatorial diameter of the bulb. The use of a smaller transducer, such as a micro-convex transducer, would probably facilitate this measurement. Despite the fact that the equipment used in our study did not produce the degree of tissue resolution obtained by some specific ophthalmic ultrasonographic devices of higher frequencies, such as 21–48 MHz [27], the images showed sufficient details for several clinical purposes. Moreover, the ultrasound machine was portable, and more accessible and affordable than non-portable ones, facilitating its use in different locations and even inside the enclosures, at the edge of the tanks where the larger animals live.

Thick scleral cartilage is related to the adaptive factors that prevent deformation of the eye bulb from water pressure during dives, and it also supports the bulb against extraocular muscle traction [4, 28, 29]. The scleral cartilage thickness in the study animals was similar to that found in other species of sea turtles by Brudenall et al. [4]. The larger measurements of the scleral ossicles found for *Caretta caretta* compared to *L. olivacea* may be assigned to the size difference of individuals between the groups. Moreover, the ossicles may not be the only factor providing protection against pressure on the eyeball, because *L. olivacea* has been reported to feed at greater depths than *Caretta caretta* [30].

The corneal thickness and the anterior chamber depth of the animals in this study were larger than what was found by Gornik et al. [10] for juvenile Kemp's ridley sea turtles (*Lepidochelys kempii*) using optical coherence tomography. Lower values for these structures were also found in frozen eyes of *Chelonia mydas*, *Dermochelys coriacea* and *E. imbricata* [31]. However, direct comparisons of values obtained using different evaluation methods should be made with caution, even for the same species, and the size of the study populations should be taken into consideration.

The sea turtle lens is more spherical in shape than that of semi-aquatic turtles [32], and this is closely related to the visual capacity of these animals [15, 32]. *Eretmochelys imbricata* had smaller lenses than the other species studied, probably because the group of *E. imbricata* had the smallest individuals in size, and a strong correlation was seen between ALL and animal size. The lens was well assessed with the equipment used in this study and it is believed that structural changes, when present, could be easily detected, even when anterior segment opacification impaired visual inspection. This is reinforced by a previous study conducted with cataracts in sea turtles that used ultrasound as an auxiliary diagnostic tool.

The AGL values were positively correlated with animal size for all species studied here. Similar correlations have been described for other reptilian species, such as tortoises, caimans and iguanas [22, 32, 33]. The lack of correlation between AGL and animal size in *L. olivacea* is believed to be due to the fact that the evaluated individuals made up a population of relatively uniform size compared to the other species.

## Conclusions

Ultrasonography proved to be practical and feasible to perform on the animals in this study, without any harmful effects on the individual, allowing indirect real-time visualization of intraocular structures. Only minor differences were found between the species in this study, reinforcing their phylogenetic proximity and their similar functions and habitats.

## Supplementary information

**Additional file 1. **Shows the data of biometric and ecobiometric values per sea turtle.

**Additional file 2: Table S1.** Shows correlation values (*r*; P-value) between body weight (BW) and curved carapace length (CCL) with ultrasound measurements in sea turtle eyes.

## Data Availability

The datasets analyzed during the current study are available from the corresponding author on reasonable request.

## Abbreviations

ACD: Anterior chamber depth
AGL: Axial globe length
ALL: Axial length of the lens
BW: Body weight
CCL: Curved carapace length
CCW: Curved carapace width
CT: Corneal thickness
MHz: Megahertz
SOT: Scleral ossicle thickness
SOW: Scleral ossicle width
UFBA: Federal University of Bahia
VC: Visitors centre
VCD: Vitreous chamber depth

## Prior publication

Data have not been published previously.
