# Supplementary material for: Ocular ultrasonography of sea turtles
Source: Acta Vet Scand. 2020 Sep 10;62:52. doi: 10.1186/s13028-020-00551-1 (PMC7488042; doi:10.1186/s13028-020-00551-1)
Supplement: Supplementary file 1 — Additional file 1. Shows the data of biometric and ecobiometric values per sea turtle. [file 13028_2020_551_MOESM1_ESM.docx]

**Additional file 1**

Biometric and ecobiometric values per sea turtle.

| Animal | BW | CCL | CCW | EYE | SOW | SOT | CT | ASD | LAL | VCD | AGL |
| --- | --- | --- | --- | --- | --- | --- | --- | --- | --- | --- | --- |
| *Caretta* *caretta* 1 | 36.2 | 69.5 | 60.6 | OD | 1.02 | 0.07 | 0.05 | 0.12 | 0.41 | 1.24 | 1.81 |
|  |  |  |  | OS | 1.07 | 0.06 | 0.05 | 0.11 | 0.46 | 1.27 | 1.86 |
| *Caretta* *caretta* 2 | 41.2 | 74.6 | 64.4 | OD | 0.96 | 0.09 | 0.04 | 0.11 | 0.41 | 1.18 | 1.64 |
|  |  |  |  | OS | 0.84 | 0.05 | 0.04 | 0.08 | 0.38 | 1.2 | 1.64 |
| *Caretta* *caretta* 3 | 27.6 | 66.6 | 55.5 | OD | 1.07 | 0.08 | 0.04 | 0.12 | 0.42 | 1.18 | 1.73 |
|  |  |  |  | OS | 1.1 | 0.06 | 0.04 | 0.08 | 0.43 | 1.18 | 1.72 |
| *Caretta* *caretta* 4 | 39.2 | 72.4 | 61.4 | OD | 1 | 0.08 | 0.04 | 0.09 | 0.4 | 1.42 | 1.9 |
|  |  |  |  | OS | 1.06 | 0.08 | 0.04 | 0.09 | 0.39 | 1.23 | 1.7 |
| *Caretta* *caretta* 5 | 162.4 | 103.6 | 89 | OD | 1.34 | 0.08 | 0.06 | 0.12 | 0.6 | 1.47 | 2.17 |
|  |  |  |  | OS | 1.18 | 0.07 | 0.06 | 0.12 | 0.57 | 1.5 | 2.19 |
| *Caretta* *caretta* 6 | 101.1 | 97.1 | 83.5 | OD | 1.18 | 0.08 | 0.05 | 0.14 | 0.54 | 1.31 | 2 |
|  |  |  |  | OS | 1.2 | 0.08 | 0.05 | 0.1 | 0.54 | 1.51 | 2.19 |
| *Caretta* *caretta* 7 | 94.4 | 91.7 | 83 | OD | 1.31 | 0.08 | 0.05 | 0.11 | 0.56 | 1.57 | 2.21 |
|  |  |  |  | OS | 1.33 | 0.1 | 0.05 | 0.14 | 0.62 | 1.57 | 2.34 |
| *Caretta* *caretta* 8 | 116 | 99.2 | 84.2 | OD | 1.43 | 0.08 | 0.05 | 0.13 | 0.64 | 1.44 | 2.2 |
|  |  |  |  | OS | 1.37 | 0.09 | 0.05 | 0.13 | 0.66 | 1.48 | 2.29 |
| *Caretta* *caretta* 9 | 106 | 93 | 82.3 | OD | 1.4 | 0.09 | 0.05 | 0.11 | 0.62 | 1.5 | 2.27 |
|  |  |  |  | OS | 1.35 | 0.09 | 0.05 | 0.14 | 0.59 | 1.52 | 2.21 |
| *Caretta* *caretta* 10 | 40.8 | 71.2 | 63.1 | OD | 0.99 | 0.08 | 0.04 | 0.09 | 0.4 | 1.24 | 1.75 |
|  |  |  |  | OS | 0.86 | 0.06 | 0.04 | 0.09 | 0.4 | 1.22 | 1.72 |
| *Chelonia mydas* 1 | 46.2 | 78.8 | 71.2 | OD | 0.91 | 0.06 | 0.04 | 0.07 | 0.5 | 1.13 | 1.7 |
|  |  |  |  | OS | 0.96 | 0.06 | 0.04 | 0.07 | 0.5 | 1.13 | 1.74 |
| *Chelonia mydas* 2 | 1.98 | 25.3 | 62.39 | OD | 0.56 | 0.05 | 0.03 | 0.07 | 0.3 | 0.68 | 1.03 |
|  |  |  |  | OS | 0.51 | 0.05 | 0.03 | 0.07 | 0.3 | 0.67 | 1.05 |
| *Chelonia mydas* 3 | 46.4 | 70.6 | 61 | OD | 0.96 | 0.07 | 0.04 | 0.11 | 0.44 | 1.02 | 1.57 |
|  |  |  |  | OS | 0.98 | 0.07 | 0.04 | 0.1 | 0.46 | 0.99 | 1.55 |
| *Chelonia mydas* 4 | 82.4 | 89 | 69.2 | OD | 1.15 | 0.07 | 0.04 | 0.2 | 0.57 | 1.21 | 1.98 |
|  |  |  |  | OS | 1.1 | 0.07 | 0.04 | 0.2 | 0.59 | 1.22 | 2.01 |
| *Chelonia mydas* 5 | 13.3 | 50 | 41 | OD | 0.89 | 0.06 | 0.04 | 0.1 | 0.42 | 0.94 | 1.47 |
|  |  |  |  | OS | 0.78 | 0.08 | 0.03 | 0.12 | 0.41 | 0.94 | 1.47 |
| *Chelonia mydas* 6 | 16.4 | 54 | 44.8 | OD | 0.85 | 0.06 | 0.04 | 0.09 | 0.4 | 0.93 | 1.39 |
|  |  |  |  | OS | 0.91 | 0.07 | 0.04 | 0.09 | 0.41 | 0.92 | 1.42 |
| *Chelonia mydas* 7 | 53.7 | 79.5 | 65.5 | OD | 1.04 | 0.07 | 0.04 | 0.12 | 0.5 | 1.08 | 1.7 |
|  |  |  |  | OS | 0.95 | 0.08 | 0.04 | 0.15 | 0.49 | 1.13 | 1.79 |
| *Chelonia mydas* 8 | 124 | 92.5 | 84 | OD | 0.99 | 0.07 | 0.04 | 0.11 | 0.54 | 1.13 | 1.78 |
|  |  |  |  | OS | 0.95 | 0.07 | 0.05 | 0.12 | 0.5 | 1.19 | 1.81 |
| *Eretmochelys imbricata* 1 | 11.3 | 48.9 | 41.2 | OD | 0.79 | 0.07 | 0.04 | 0.08 | 0.36 | 0.96 | 1.4 |
|  |  |  |  | OS | 0.71 | 0.07 | 0.04 | 0.09 | 0.35 | 0.91 | 1.34 |
| *Eretmochelys imbricata* 2 | 11.7 | 47.3 | 41.6 | OD | 0.7 | 0.07 | 0.04 | 0.08 | 0.34 | 0.92 | 1.35 |
|  |  |  |  | OS | 0.78 | 0.07 | 0.04 | 0.08 | 0.35 | 0.95 | 1.4 |
| *Eretmochelys imbricata* 3 | 11.7 | 46.5 | 41.5 | OD | 0.76 | 0.07 | 0.04 | 0.09 | 0.36 | 0.97 | 1.41 |
|  |  |  |  | OS | 0.81 | 0.07 | 0.03 | 0.08 | 0.36 | 0.98 | 1.42 |
| *Eretmochelys imbricata* 4 | 14.7 | 52.1 | 45.1 | OD | 0.85 | 0.07 | 0.03 | 0.08 | 0.36 | 1 | 1.44 |
|  |  |  |  | OS | 0.91 | 0.06 | 0.04 | 0.17 | 0.48 | 1.18 | 1.82 |
| *Eretmochelys imbricata* 5 | 52.8 | 77 | 68 | OD | 0.99 | 0.07 | 0.04 | 0.16 | 0.47 | 1.16 | 1.79 |
|  |  |  |  | OS | 0.96 | 0.06 | 0.04 | 0.1 | 0.46 | 1.14 | 1.7 |
| *Eretmochelys imbricata* 6 | 32 | 67.7 | 57 | OD | 1.04 | 0.07 | 0.04 | 0.09 | 0.46 | 1.15 | 1.72 |
|  |  |  |  | OS | 0.8 | 0.06 | 0.04 | 0.08 | 0.36 | 0.96 | 1.39 |
| *Eretmochelys imbricata* 7 | 11.4 | 47.6 | 41.5 | OD | 0.78 | 0.05 | 0.04 | 0.09 | 0.37 | 0.95 | 1.41 |
|  |  |  |  | OS | 1.22 | 0.06 | 0.04 | 0.09 | 0.53 | 1.21 | 1.82 |
| *Eretmochelys imbricata* 8 | 69 | 84 | 68.7 | OD | 0.67 | 0.05 | 0.04 | 0.07 | 0.32 | 0.97 | 1.39 |
|  |  |  |  | OS | 0.80 | 0.06 | 0.04 | 0.09 | 0.37 | 0.96 | 1.43 |
| *Lepidochelys olivacea* 1 | 38.6 | 68.3 | 64.3 | OD | 1.04 | 0.07 | 0.04 | 0.11 | 0.54 | 1.25 | 1.9 |
|  |  |  |  | OS | 1 | 0.06 | 0.04 | 0.11 | 0.55 | 1.26 | 1.9 |
| *Lepidochelys olivacea* 2 | 34 | 64.3 | 65.4 | OD | 0.96 | 0.06 | 0.04 | 0.1 | 0.53 | 1.18 | 1.82 |
|  |  |  |  | OS | 1.04 | 0.06 | 0.04 | 0.12 | 0.5 | 1.2 | 1.82 |
| *Lepidochelys olivacea* 3 | 46.8 | 69.1 | 69.2 | OD | 1.07 | 0.07 | 0.04 | 0.09 | 0.59 | 1.23 | 1.91 |
|  |  |  |  | OS | 1.07 | 0.08 | 0.04 | 0.09 | 0.58 | 1.24 | 1.92 |
| *Lepidochelys olivacea* 4 | 30 | 58.5 | 57.6 | OD | 1.04 | 0.07 | 0.06 | 0.13 | 0.55 | 1.2 | 1.89 |
|  |  |  |  | OS | 1.03 | 0.07 | 0.06 | 0.13 | 0.52 | 1.2 | 1.87 |
| *Lepidochelys olivacea* 5 | 36 | 60.2 | 58.5 | OD | 1.14 | 0.06 | 0.05 | 0.14 | 0.53 | 1.28 | 1.94 |
|  |  |  |  | OS | 1.1 | 0.06 | 0.05 | 0.09 | 0.55 | 1.24 | 1.9 |
| *Lepidochelys olivacea* 6 | 20 | 55 | 63 | OD | 1.05 | 0.06 | 0.04 | 0.13 | 0.5 | 1.16 | 1.79 |
|  |  |  |  | OS | 0.98 | 0.06 | 0.05 | 0.13 | 0.5 | 1.18 | 1.79 |

BW, body weight; CCL, curved carapace length; CCW, curved carapace width; SOW, scleral ossicle width; SOT, scleral ossicle thickness; CT, corneal thickness; ASD, anterior chamber depth; LAL, lens axial length; VCD, vitreous chamber depth; AGL, axial globe length; OD, right eye; OS, left eye.
