# Supplementary material for: Ocular ultrasonography of sea turtles
Source: Acta Vet Scand. 2020 Sep 10;62:52. doi: 10.1186/s13028-020-00551-1 (PMC7488042; doi:10.1186/s13028-020-00551-1)
Supplement: Supplementary file 2 — Additional file 2: Table S1. Shows correlation values (r; P-value) between body weight (BW) and curved carapace length (CCL) with ultrasound measurements in sea turtle eyes. [file 13028_2020_551_MOESM2_ESM.docx]

**Table S1** Correlation values (*r*; *P-Value*) between body weight (BW) and curved carapace length (CCL) with ultrasound measurements in sea turtle eyes.

|  |  | **All animals** | ***Caretta caretta*** | ***Chelonia* *mydas*** | ***Eretmochelys imbricata*** | ***Lepidochelys olivacea*** |
| --- | --- | --- | --- | --- | --- | --- |
| **BW** | BW | - | - | - | - | - |
|  | CCL | 0.976; 0.000 | 0.976; 0.000 | 0.976; 0.000 | 0.778; 0.000 | 0.943; 0.000 |
| **CCW** | BW | 0.953; 0.000 | 0.964; 0.000 | 0.857; 0.000 | 0.982; 0.000 | 0.600; 0.039 |
|  | CCL | 0.933; 0.000 | 0.976; 0.000 | 0.929; 0.000 | 0.814; 0.000 | 0.771; 0.003 |
| **SOW** | BW | 0.734; 0.000 | 0.674; 0.001 | 0.848; 0.000 | 0.839; 0.000 | 0.414; 0.181 |
|  | CCL | 0.732; 0.000 | 0.657; 0.002 | 0.815; 0.000 | 0.871; 0.000 | 0.185; 0.564 |
| **SOT** | BW | 0.489; 0.000 | 0.420; 0.065 | 0.562; 0.023 | 0.023; 0.931 | 0.411; 0.184 |
|  | CCL | 0.483; 0.000 | 0.404; 0.077 | 0.485; 0.057 | -0.302; 0.255 | 0.411; 0.184 |
| **CT** | BW | 0.509; 0.000 | 0.724; 0.000 | 0.734; 0.001 | -0.083; 0.760 | -0.478; 0.116 |
|  | CCL | 0.504; 0.000 | 0.724; 0.000 | 0.734; 0.001 | -0.082; 0.761 | -0.637; 0.026 |
| **ACD** | BW | 0.538; 0.000 | 0.526; 0.017 | 0.690; 0.003 | 0.663; 0.005 | -0.697; 0.012 |
|  | CCL | 0.503; 0.000 | 0.519; 0.019 | 0.618; 0.011 | 0.481; 0.059 | -0.741; 0.006 |
| **ALL** | BW | 0.777; 0.000 | 0.719; 0.000 | 0.869; 0.000 | 0.793; 0.000 | 0.797; 0.002 |
|  | CCL | 0.719; 0.000 | 0.684; 0.001 | 0.916; 0.000 | 0.827; 0.000 | 0.697; 0.012 |
| **VCD** | BW | 0.721; 0.000 | 0.675; 0.001 | 0.865; 0.000 | 0.823; 0.000 | 0.757; 0.004 |
|  | CCL | 0.728; 0.000 | 0.669; 0.001 | 0.913; 0.000 | 0.913; 0.000 | 0.571; 0.052 |
| **AGL** | BW | 0.772; 0.000 | 0.656; 0.002 | 0.895; 0.000 | 0.857; 0.000 | 0.843; 0.001 |
|  | CCL | 0.745; 0.000 | 0.630; 0.003 | 0.927; 0.000 | 0.858; 0.000 | 0.657; 0.020 |

CCW. curved carapace width; SOW. scleral ossicle width; SOT. scleral ossicle thickness; CT. corneal thickness; ACD. anterior chamber depth; ALL. axial length of the lens; VCD. vitreous chamber depth; AGL. axial globe length.
